# Tetradentate copper complex supported on boehmite nanoparticles as an efficient and heterogeneous reusable nanocatalyst for the synthesis of diaryl ethers

**DOI:** 10.1038/s41598-022-15921-0

**Published:** 2022-07-08

**Authors:** Arida Jabbari, Parisa Moradi, Maryam Hajjami, Bahman Tahmasbi

**Affiliations:** 1 Department of Chemistry, Qeshm Branch, Islamic Azad University, Qeshm, Iran; 2 Department of Chemistry, Ilam University, P.O. Box 69315516, Ilam, Iran; 3 Department of Organic Chemistry, Faculty of Chemistry, Bu-Ali Sina University, Hamedan, 6517838683 Iran

**Keywords:** Chemistry, Nanoscience and technology

## Abstract

In this work boehmite nanoparticles (BNPs) were prepared through addition of aqueous solution of NaOH to solution of Al(NO_3_)_3_·9H_2_O. Then, the surface of BNPs was modified by (3-chloropropyl)trimethoxysilane (CPTMS) and further tetradentate ligand (MP-bis(AMP)) was anchored on its surface. At final step, a tetradentate organometallic complex of copper was stabilized on the surface of modified BNPs (Cu(II)-MP-bis(AMP)@boehmite). These obtained nanoparticles were characterized using SEM imaging, WDX, EDS, AAS and TGA analysis, BET method, FT-IR spectroscopy, and XRD pattern. In continue, the catalytic activity of Cu(II)-MP-bis(AMP)@boehmite has been used as a much efficient, reusable and hybrid of organic–inorganic nanocatalyst in the synthesis of ether derivatives through C–O coupling reaction under palladium-free and phosphine-free conditions. Cu(II)-MP-bis(AMP)@boehmite catalyst has been recovered and reused again for several times in the synthesis of ether derivatives.

## Introduction

The field of nanoparticles solid-state nanoparticles have been grown strong consideration in green chemistry, biological applications and scientific research due to their inimitable properties such as excellent particular surface area, high stability, non-toxicity and high environmental friendly^1–5^. For example, polymers^6^, carbon nanotubes^7^, MCM-41^8,9^, ionic liquids^10^, boehmite^11,12^, zeolite^13^, biochar^14,15^, graphene oxide^16^, magnetic nanoparticles^17–20^, and etc. were demonstrated in variant fields especially in catalysis knowledge. Amongst them, BNPs encompass variant usages such as the vaccine adjuvants, optical material, photoluminescent substance, flame retardant, plastics reinforcing, coatings, composite material amplification in ceramics, support for catalysts, starting substance in the alumina synthesis and so on^12,21–28^. BNPs are one of the polymorph of aluminum oxide hydroxide (γ‐AlOOH) that are form twice sheet construction and composed just aluminum and oxygen^29,30^. The surface of BNPs encompass high aggregation of OH groups, which makes possible its surface modification with various functional groups to stabilization of catalysts^31^. Therefore recently, BNPs were synthesized by variant procedure and peculiarly used as support for catalysts^12^. BNPs were generally formed by hydrolysis of Al-salts such as NaAlO_2_, Al_2_(SO_4_)_3_, AlCl_3_, Al(NO_3_)_3_, or aluminum alkoxide^32–37^. Herein, variant processes have been introduced for synthesis of BNPs such as hydrothermal^38^, solvothermal^30^, sol–gel^39^, coprecipitation^40^ reactions and hydrolysis of aluminum^41^. Stability, availability and environmentally of BNPs are significant benefits for usage in industrial and academic research^42,43^.

Carbon–oxygen coupling reaction as powerful tools for the synthesis of ether derivatives is generally offered with Pd-catalysts containing phosphine ligands^44–47^. The use of palladium catalyst and phosphine ligands led to expensive, toxic and also air or moisture sensitive of procedure. More addition, instability and non‐recoverability are major drawback of homogeneous catalysts and phosphine materials^16^. Meanwhile, copper catalyst without phosphine ligands is non-toxic and inexpensive than palladium catalyst and high environmentally friendly and moisture- or air-stability^16,48^. Therefore, to make pace principles of green chemistry, we investigated Cu-catalyst on BNPs as a stable and recyclable nanocatalyst for the C–O coupling reaction.

## Result and discussion

**Figure 1:**
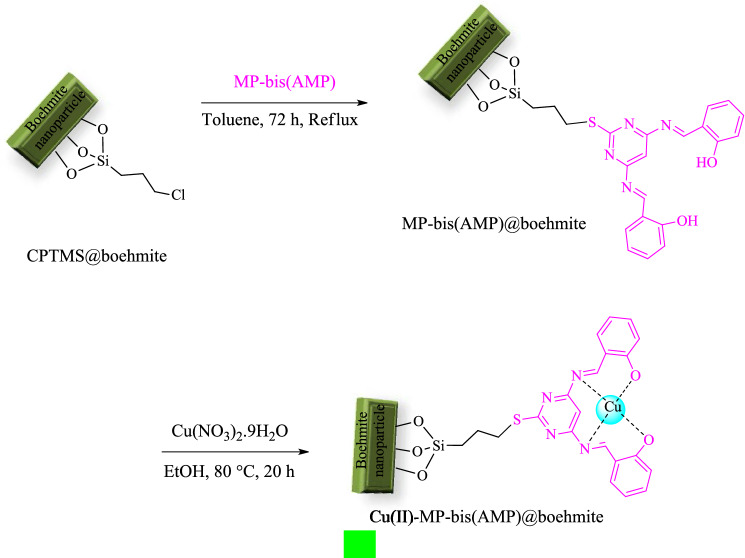
Synthesis of Cu(II)-MP-bis(AMP)@boehmite.

Surface modification of BNPs by CPTMS was performed matching to last reported method^15^. Then, MP-bis(AMP) ligand was substituted with Cl of CPTMS. Subsequently copper catalyst was fixated on the surface. The schematic preparation procedure of this catalyst (Cu(II)-MP-bis(AMP)@boehmite) is outlined in Fig. 1.

**Figure 2:**
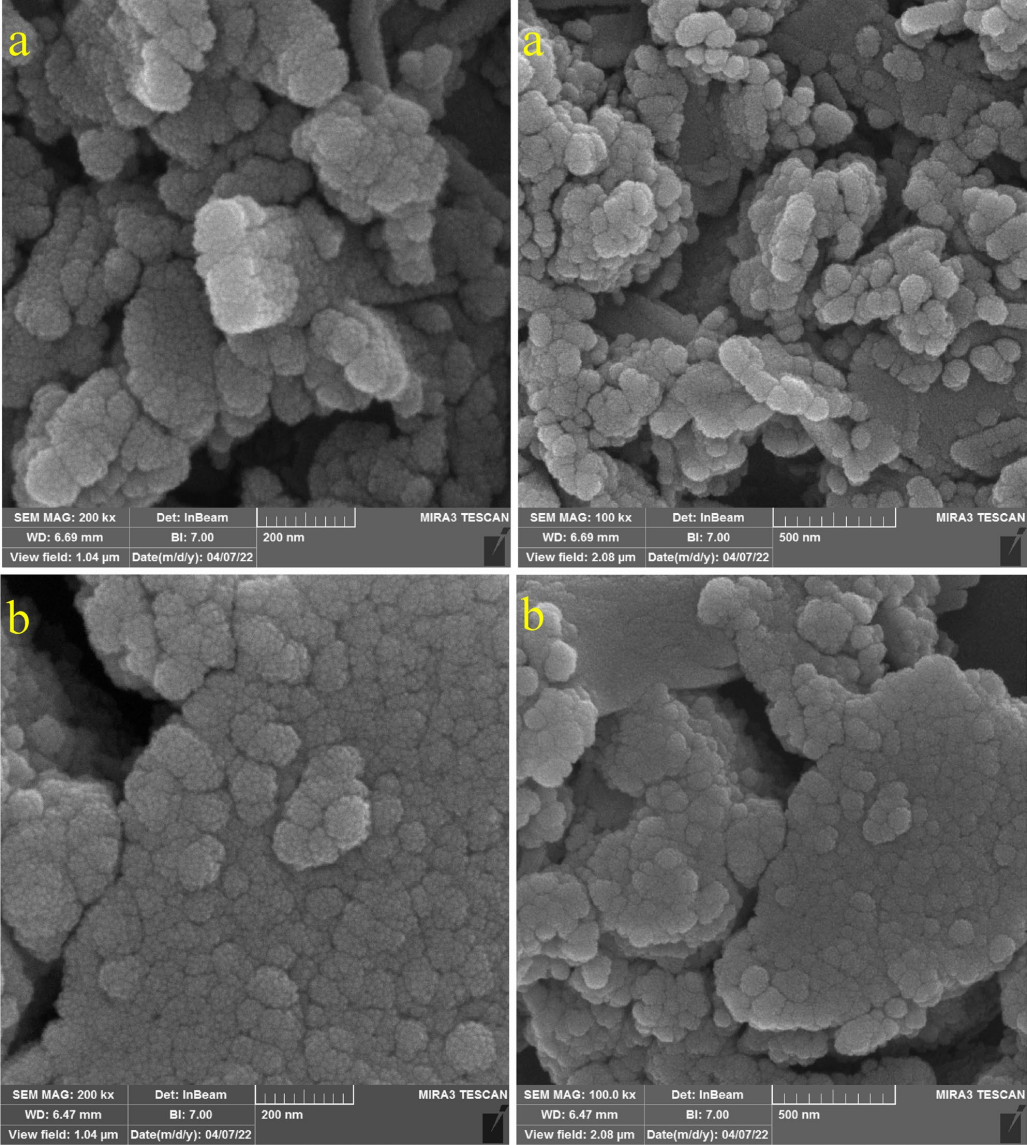
SEM image of (**a**) Cu(II)-MP-bis(AMP)@boehmite and (**b**) MP-bis(AMP)@boehmite.

Cu(II)-MP-bis(AMP)@boehmite was characterized by scanning electron microscopy (SEM) imaging, wavelength dispersive X-ray spectroscopy (WDX), energy-dispersive X-ray spectroscopy (EDS), atomic absorption spectroscopy (AAS) and thermogravimetric analysis (TGA) analysis, X-ray diffraction (XRD) pattern, Fourier transform infrared spectroscopy (FT-IR) spectroscopy, N_2_ adsorption–desorption isotherms method. SEM images of Cu(II)-MP-bis(AMP)@boehmite and MP-bis(AMP)@boehmite are shown in Fig. 2a,b respectively which indicate that Cu(II)-MP-bis(AMP)@boehmite has particle size in nanometer scale. As shown in Fig. 2, the SEM images of the material before the addition of Cu are similar to after the addition of Cu in term of size and morphology which shows the stability of these nanoparticles after stabilization of the copper complex.

**Figure 3:**
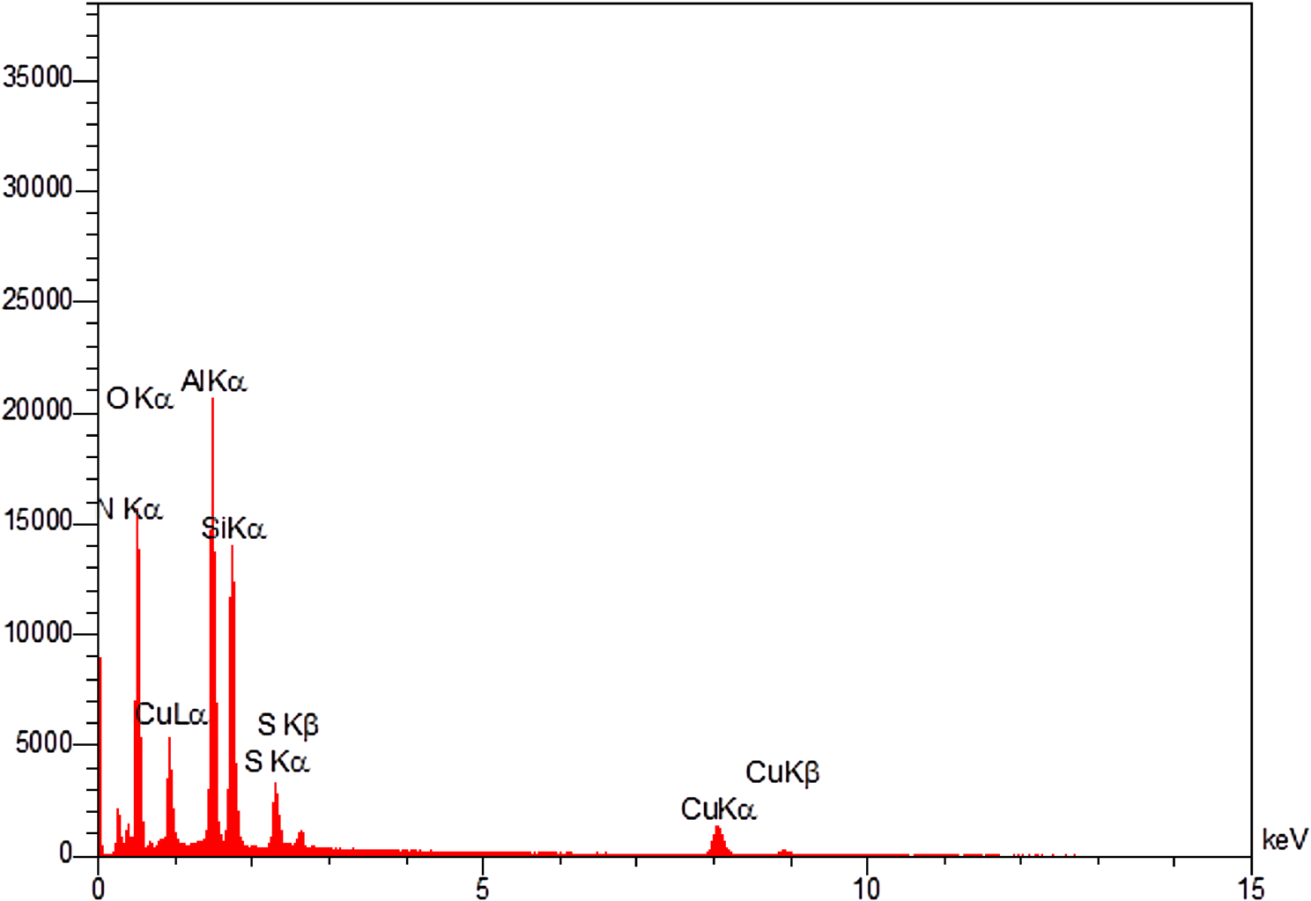
EDS diagram of Cu(II)-MP-bis(AMP)@boehmite.

**Figure 4:**
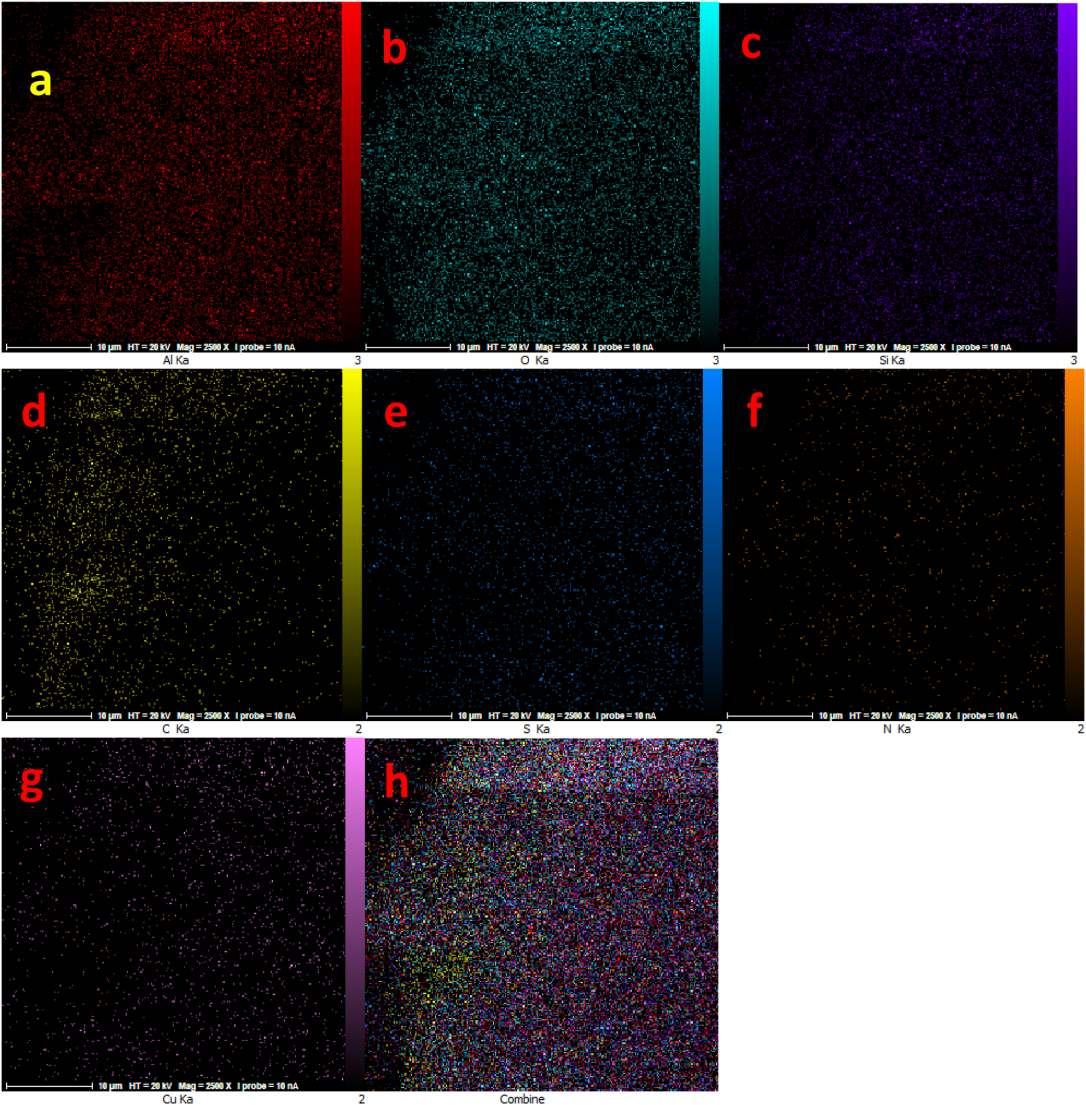
Elemental mapping of (**a**) aluminum, (**b**) oxygen, (**c**) silica, (**d**) carbon, (**e**) sulfur, (**f**) nitrogen, (**g**) copper and combine elements for Cu(II)-MP-bis(AMP)@boehmite.

In order to illustrate the elemental combination and distributions of catalyst, the energy-dispersive X-ray spectroscopy (EDS) and wavelength-dispersive X-ray mapping (WDX) analysis of Cu(II)-MP-bis(AMP)@boehmite have been examined, the EDS (Fig. 3) and WDX (Fig. 4) analysis of this catalyst shown the attendance of aluminum, oxygen, silica, carbon, sulfur, nitrogen, and in addition copper species in catalyst with homogeneous dispensations of all elements in the structure of Cu(II)-MP-bis(AMP)@boehmite. Also, the exact amount of copper was found to be 0.4 × 10^−3^ mol g^−1^ by AAS analysis.

**Figure 5:**
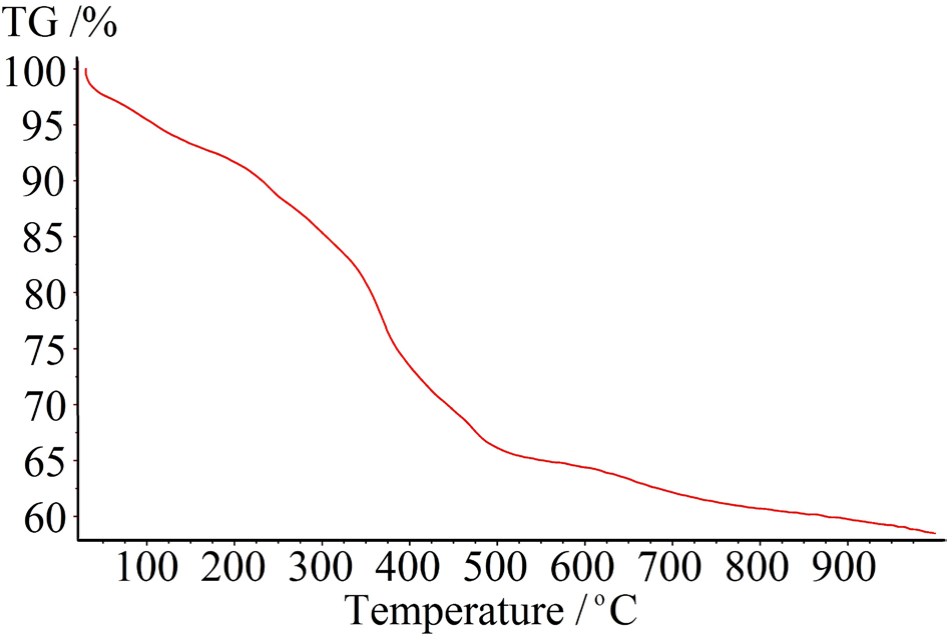
TGA/DTA diagrams of Cu(II)-MP-bis(AMP)@boehmite.

In order to determine content of organic species, which were immobilized on the surface of BNPs, TGA/DTA analysis of Cu(II)-MP-bis(AMP)@boehmite was performed (Fig. 5). The miniature weight loss within 9% at downward temperature is related to vaporization of adsorbed solvents^49^. The organic substance including CPTMS and ligand which fixed on BNPs was decomposed at 200–500 °C that is 32% of catalyst. Last weight dissipation which is lesser than 2% a may be related to transformation of thermal crystal phase of boehmite nanoparticles^11^.

**Figure 6:**
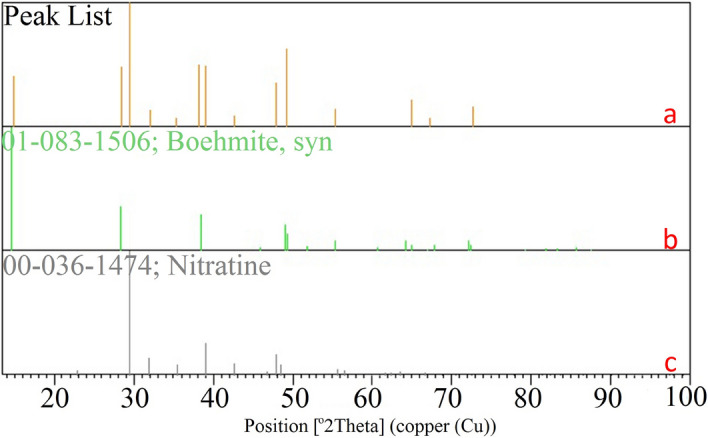
XRD pattern of Cu(II)-MP-bis(AMP)@boehmite (**a**), standard pattern 01-083-1506 code of boehmite (**b**) and standard pattern 00-036-1474 code of sodium nitrate (**c**).

Powder XRD analysis is a great technique to determine the crystal structure of materials. Therefore, the powder XRD analysis was performed to shown the crystalline phase of Cu(II)-MP-bis(AMP)@boehmite. The experimentally obtained XRD patterns were compared to the Inorganic Crystal Structure Database (ICSD) provided which shows two series of crystal structure. The obtained results analysis from powder XRD analysis of Cu(II)-MP-bis(AMP)@boehmite is shown in Fig. 6. Also, phases list from XRD results are summarized in Table 1. As shown in Fig. 5 and Table 1, X-ray diffraction analysis of this catalyst shows two series of materials. The first of them is related to the boehmite (Aluminum Oxide Hydroxide) crystal phase, which matched with the standard pattern 01-083-1506 code of ICSD database. This pattern correspond to 2θ value positions at 14.8° (0 2 0), 28.48° (1 2 0), 38.27° (0 3 1), 46.45° (1 3 1), 49.24° (0 5 1), 51.94° (2 0 0), 55.49° (1 5 1), 59.35° (0 8 0), 64.91° (2 3 1), 65.56° (0 0 2), 67.23° (1 7 1), and 72.65° (2 5 1), which are consistent with the orthorhombic unit cell of standard pattern of boehmite nanoparticles^50^. Therefore, the initial boehmite phase is stable during the boehmite modification and copper stabilization on its surface. The second pattern is related to the sodium nitrate as impurity (which matched with the standard pattern 00-036-1474 code of ICSD database). This pattern corresponds to 2θ value positions at 29.43°, 32.04°, 35.39°, 39.04°, 62.63°, 47.94°, 55.49°, 67.23° and 72.65°. This sodium nitrate impurity is also commonly seen in the IR spectrum in region 1650 cm^−1^. As shown, some peaks of the sodium nitrate crystal phase pattern overlap with the boehmite crystal phase peaks at 2θ value positions 32.04°, 39.04°, 47.94°, 55.49°, 67.23° and 72.65°.

**Table 1:** Identified patterns list from XRD results of Cu(II)-MP-bis(AMP)@boehmite catalyst.

Visible	Ref. code	Score	Compound name	Displacement [°2Th.]	Scale factor	Chemical formula
*	01-083-1506	77	Aluminum oxide hydroxide	0.191	0.774	AlO(OH)
*	00-036-1474	82	Sodium nitrate	0.066	1.128	NaNO_3_

**Figure 7:**
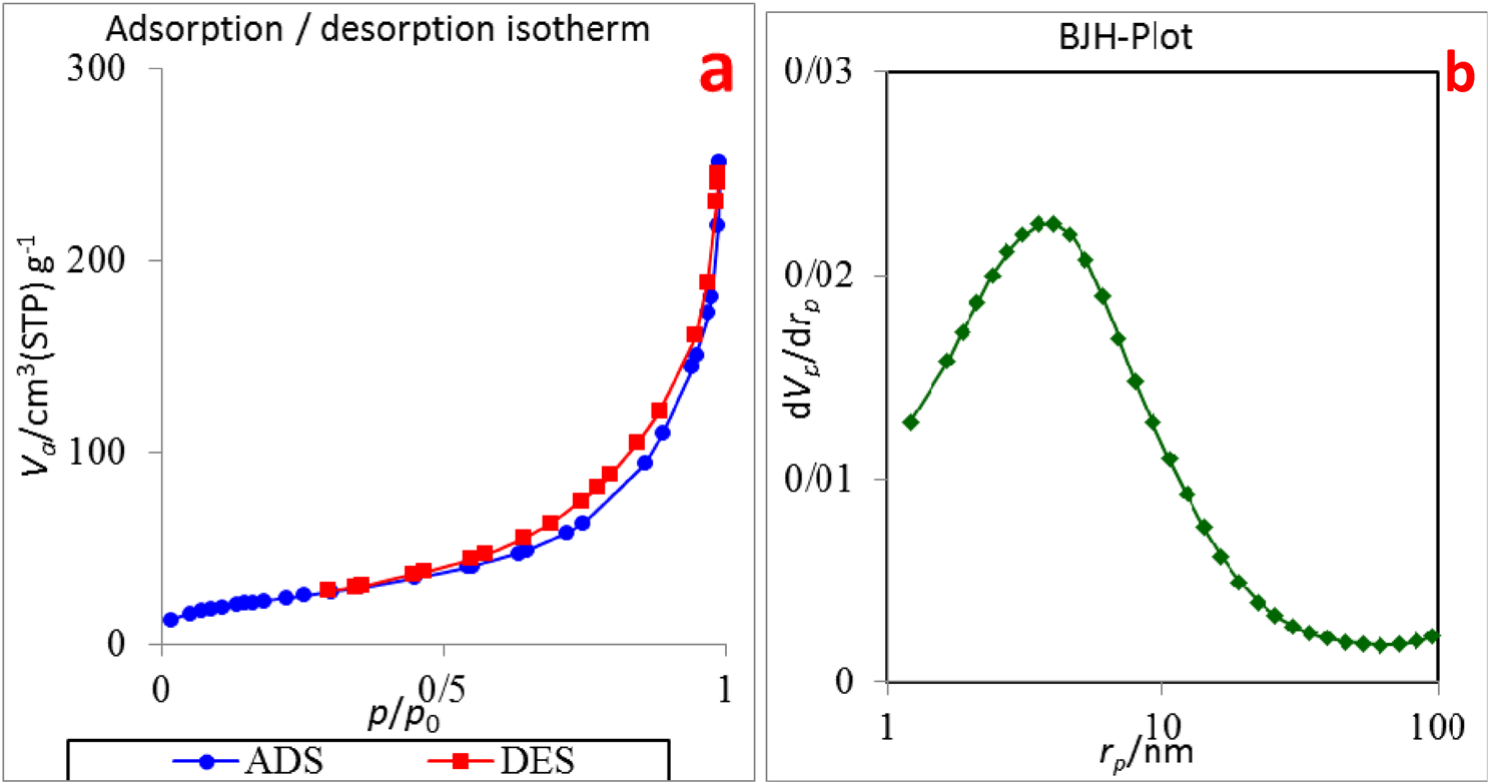
(**a**) N_2_ adsorption–desorption isotherm and (**b**) BJH-Plot of Cu(II)-MP-bis(AMP)@boehmite.

The N_2_ adsorption–desorption isotherms and BJH-Plot diagram of Cu(II)-MP-bis(AMP)@boehmite are shown in Fig. 7 and textural properties of Cu(II)-MP-bis(AMP)@boehmite are summarized in Table 2. As shown in Table 2, surface area, pore volumes and pore diameters of this catalyst are 101.66 m^2^ g^−1^, 0.375 cm^3^ g^−1^ and 4.62 nm respectively. Decreasing of surface area of Cu(II)-MP-bis(AMP)@boehmite than boehmite nanoparticles (128.8 m^2^ g^−1^, Ref.^29^) is due to the linking of organic substance and copper complex.

**Table 2:** Textural properties of Cu(II)-MP-bis(AMP)@boehmite.

S_BET_ (m^2^ g^−1^)	Pore diameter (nm)	Pore volume (cm^3^ g^−1^)
101.66	4.62	0.375

**Figure 8:**
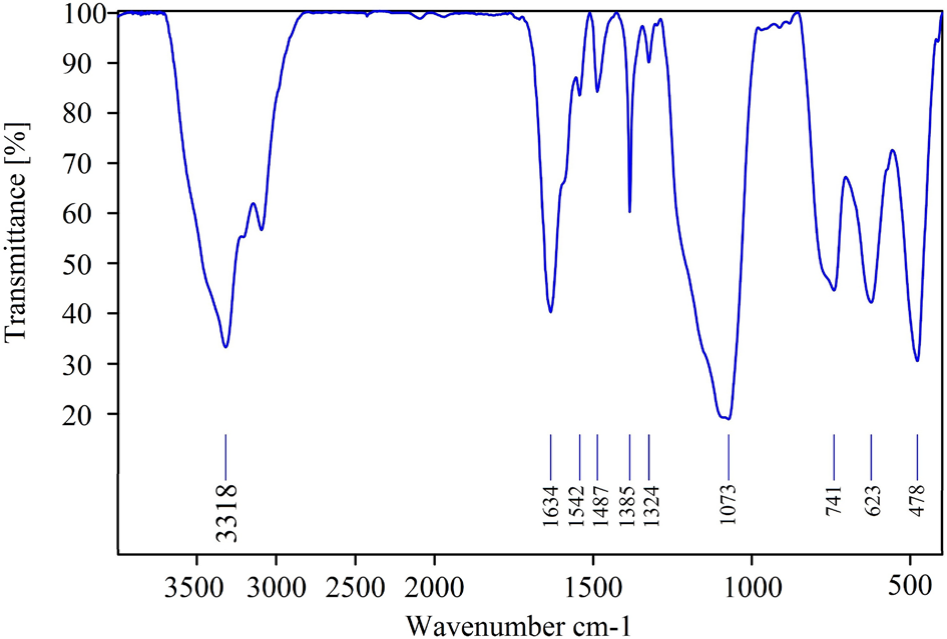
FT-IR spectrum of Cu(II)-MP-bis(AMP)@boehmite.

The FT-IR spectrum of Cu(II)-MP-bis(AMP)@boehmite is illustrated in Fig. 8. Also, FT-IR characteristic absorptions of Cu(II)-MP-bis(AMP)@boehmite are summarized in Table 3. The FT-IR spectrum of Cu(II)-MP-bis(AMP)@boehmite is demonstrated a strong peak at 1634 cm^−1^, which are related to the C=N vibrations in the structure of the fabricated ligand^15^. The several peaks which are shown in region < 3000 cm^−1^ related to the vibrations of the C–H bonds of immobilized organic groups on the surface of boehmite nanoparticles^51^. The stretching vibration of Si–O is observed at 1073 cm^−1^^51^. The stretching vibration of hydroxyl groups in FT-IR spectrum of Cu(II)-MP-bis(AMP)@boehmite is appeared at 3318 cm^−1^^51^. The vibrations of hydrogen bands of OH⋯OH is indicated at 1164 cm^−1^^52^. Also, the bending vibration of hydroxyl groups is appeared at 1385 cm^−1^^15^. The three bands at 478, 623 and 741 cm^−1^ are referred to the vibration of the Al–O bonds in boehmite nanoparticles^52^. The characteristic of the NaNO_3_ impurity was emerged at 1650 cm^−1^^52^ which overlap with the vibrations of the other bonds. This impurity is also observed in the XRD analysis.

**Table 3:** FT-IR characteristic absorption of Cu(II)-MP-bis(AMP)@boehmite.

Entry	Functional group	Absorption (cm^−1^)	References
1	C–H stretch	< 3000	^51^
2	O–H stretch	3318	^51^
3	O–H bending	1385	^15^
4	C=N stretch	1634	^15^
5	Si–O	1073	^51^
6	Al–O	478, 623, 741	^52^
7	OH⋯OH	1164	^52^
8	NaNO_3_	1650	^52^

### Catalytic study of Cu(II)-MP-bis(AMP)@boehmite

**Scheme 1:**
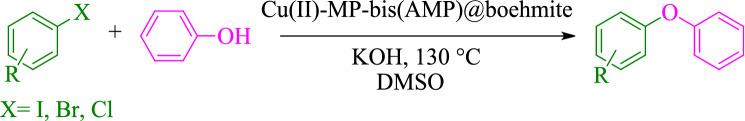
Synthesis of diaryl ether derivatives using Cu(II)-MP-bis(AMP)@boehmite.

The catalytic activity of Cu(II)-MP-bis(AMP)@boehmite has been investigated in the C-O coupling reaction toward the formation of diaryl ether derivatives. In the synthesis of diaryl ethers, the coupling of phenol (Ph-OH) with iodobenzene (Ph-I) using catalytic value of Cu(II)-MP-bis(AMP)@boehmite as catalyst has been chosen as a pattern reaction to found the optimize conditions. At first, the pattern reaction has been tested without Cu(II)-MP-bis(AMP)@boehmite (Table 4, entry 1) which the pattern reaction was not go proceed. Then, the pattern reaction was carried out in using variant value of catalyst which it was completed with 98% of yield when 30 mg of Cu(II)-MP-bis(AMP)@boehmite was used (Table 4, entry 2). At second, the effect of various solvents (Table 4, entries 4–7) and bases (Table 4, entries 8–11) were studied in the pattern reaction under wide range of temperature. As shown, DMSO solvent and KOH base at 130 ºC offered the best results for the synthesis of diaryl ether (Scheme 1).

**Table 4:** Optimizing conditions for the synthesis of diaryl ether using Cu(II)-MP-bis(AMP)@boehmite.

Entry	Catalyst(mg)	Solvent	Base	Temperature (°C)	Time (min)	Yield^a^ (%)
1	–	DMSO	KOH	130	300	N.R^b^
2	30	DMSO	KOH	130	60	98
3	20	DMSO	KOH	130	100	93
4	35	DMSO	KOH	130	55	95
5	30	PEG-400	KOH	130	60	38
6	30	DMF	KOH	130	60	69
7	30	H_2_O	KOH	Reflux	60	30
8	30	DMSO	Na_2_CO_3_	130	60	22
9	30	DMSO	NaHCO_3_	130	60	31
10	30	DMSO	Et_3_N	130	60	48
11	30	DMSO	NaOH	130	60	82
12	30	DMSO	KOH	100	60	65

^a^Isolated yield.

^b^No reaction.

In order to show the role of Cu(II)-MP-bis(AMP)@boehmite, the catalytic activity of Cu(II)-MP-bis(AMP)@boehmite was compared with alone boehmite and MP-bis(AMP)@boehmite in the coupling of phenol with iodobenzene under optimized conditions (Table 5). As shown, diphenyl ether was formed in the presence of Cu(II)-MP-bis(AMP)@boehmite with 98% of yield. While, almost no products were formed in the presence of alone boehmite or MP-bis(AMP)@boehmite.

**Table 5:** A comparison of Cu(II)-MP-bis(AMP)@boehmite with boehmite or MP-bis(AMP)@boehmite as catalyst in the coupling of phenol with iodobenzene under optimized conditions.

Entry	Catalyst	Time (h)	Yield (%)^a^
1	Boehmite nanoparticles	1	Trace
2	MP-bis(AMP)@boehmite	1	Trace
3	Cu(II)-MP-bis(AMP)@boehmite	1	98

^a^Isolated yield. Catalyst (30 mg) and KOH (5 mmol) in DMSO at 130 °C.

The mentioned optimizing condition were investigated to the various aryl halide derivatives to extend catalytic scope of Cu(II)-MP-bis(AMP)@boehmite (Table 6). All aryl halide derivatives having other functional groups with electron-withdrawing or electron-donating nature were successfully coupled with phenol in superior yields in the presence of this catalyst. As shown in Table 6, aryl iodides have great reaction rate than aryl bromides, while aryl chlorides have lowest reaction rate under coupling of phenol using Cu(II)-MP-bis(AMP)@boehmite catalyst. This indicates that the C–Cl bond is stronger than the C–I bond because the carbon and chlorine orbitals are similar in size, energy, and symmetry, but the iodine and carbon orbitals have different sizes and energies. In addition, the C–I bond is longer and weaker than the C–Cl bond, which C–I bond requires less energy to break and has a faster coupling rate than the short C–Cl bond. For example, the coupling of phenol with 4-nitrobromobenzene is greater than 4-nitrochlorobenzene. This ordered was also observed at coupling of phenol with iodobenzene, bromobenzene and chlorobenzene using Cu(II)-MP-bis(AMP)@boehmite catalyst.

**Table 6:** Synthesis of diaryl ether derivatives using Cu(II)-MP-bis(AMP)@boehmite.

Entry	Aryl halide	Product	Time (h)	Yield (%)
			1	98
			3.05	95
			11.30	94
			4.05	95
			3.35	87
			10	92
			2.30	94
			4	86
			15	84
			5	91

Isolated yield.

### Comparison of the catalyst

The activity and practicality of Cu(II)-MP-bis(AMP)@boehmite catalyst in comparison with reported catalysts in the literatures are listed in Table 6 for coupling of Ph-OH with Ph-I. As monitored in Table 7, biphenyl ether was synthesized in superior yields when Cu(II)-MP-bis(AMP)@boehmite employed as catalyst than other catalysts. Therefore, Cu(II)-MP-bis(AMP)@boehmite catalyst is more effective than alternative catalysts in terms of practicality, reaction rate and isolated yield. Also, in some cases, non-recoverable homogeneous catalysts have been introduced for the formation of aromatic ethers (Table 7, entry 7). While, Cu(II)-MP-bis(AMP)@boehmite catalyst can be recycled over and over again.

**Table 7:** Comparison results of Cu(II)-MP-bis(AMP)@boehmite catalyst with other reported catalysts in the synthesis of biphenyl ether.

Entry	Catalyst	Reaction conditions	Time (h)	Yield (%) (References)
1	CuNPs/CNFs	CsCO_3_, DMAc, 140 °C	24	100^53^
2	CuO nanoparticles	KOH, DMSO, 110 °C, N_2_ atmosphere	15	93^54^
3	Cu nanoparticles	CsCO_3_, CH_3_CN, 50–60 °C, N_2_ atmosphere	4	91^55^
4	Fe_3_O_4_/CS-Cu	K_2_CO_3_, DMSO, 120 °C	15	95^56^
5	Cu/RGO-Fe_3_O_4_ Cu nanoparticles	CsCO_3_, DMSO, 120 °C	12	98^57^
6	Cu_2_O/SiC	CsCO_3_, THF, 150 °C	3	97^58^
7	CuI	^n^Bu_4_NBr, K_3_PO_4_, DMF, Reflux	22	95^59^
8	Natural ferrous chamosite	K_2_CO_3_, DMF, 110 °C	12	97^60^
9	Cu–ninhydrin@GO–Ni MNPs	KOH, DMSO, 130 °C	2	98^16^
10	Cu(II)-MP-bis(AMP)@boehmite	KOH, DMSO, 130 °C	1	98 [This work]

### Recycling ability and leaching study of the catalyst

**Figure 9:**
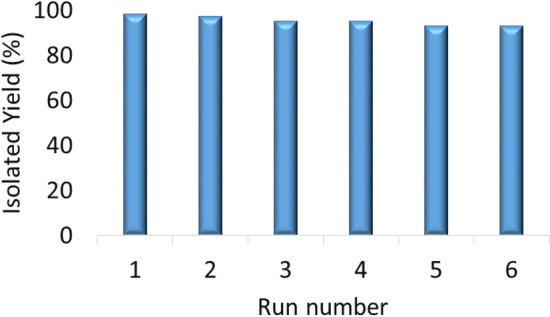
Recyclability of Cu(II)-MP-bis(AMP)@boehmite in the coupling of phenol with iodobenzen.

Cu(II)-MP-bis(AMP)@boehmite catalyst can be isolated easily by centrifugation and recycled again for multifold times. For this issue, recoverability of Cu(II)-MP-bis(AMP)@boehmite was investigated in the coupling of Ph-O with Ph-I. At first, coupling of Ph-O with Ph-I was started under optimized conditions and after termination of the reaction, the residue catalyst was separated via centrifugation. Then, the divided catalyst was washed and then it was employed again in the next run for 6 cycles. As shown in Fig. 9, Cu(II)-MP-bis(AMP)@boehmite catalyst can be recycled frequently at minimum to 6 times in synthesis of biphenyl ether.

The copper leaching from Cu(II)-MP-bis(AMP)@boehmite in the reaction mixture was studied by AAS analysis. In order to this issue, the coupling reaction of Ph-O with Ph-I in the presence of Cu(II)-MP-bis(AMP)@boehmite was repeated and the catalyst was recovered and collected after completion of the reaction. Then, the amount of copper in the recovered catalyst (0.32 × 10^−3^ mol g^−1^) was compared with the unused catalyst (0.4 × 10^−3^ mol g^−1^) by AAS analysis which indicated that copper leaching of this catalyst is negligible (less than 0.01%).

**Figure 10:**
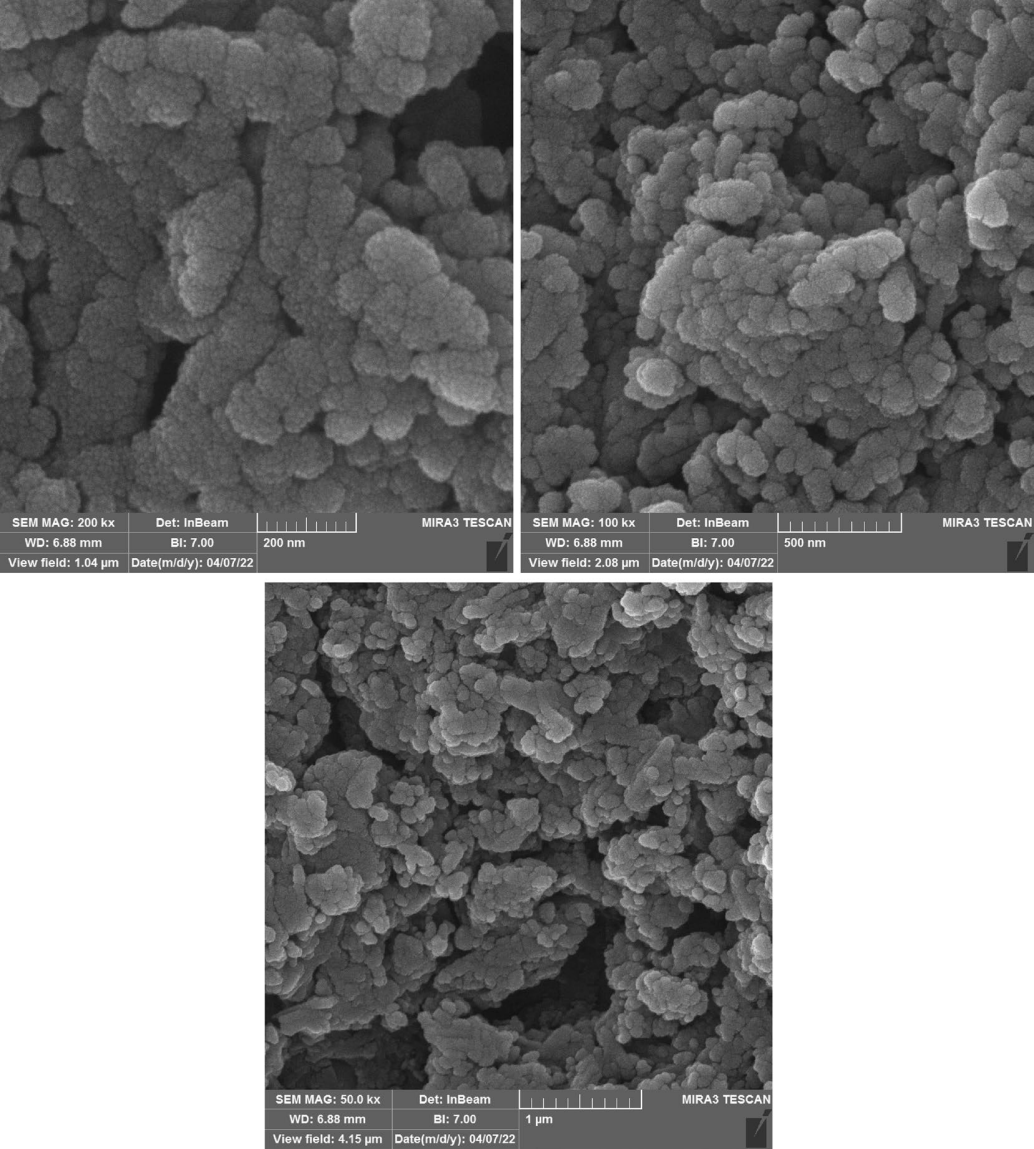
SEM images of Cu(II)-MP-bis(AMP)@boehmite after recovery.

The SEM images of Cu(II)-MP-bis(AMP)@boehmite after recovered and reused are shown in Fig. 10. The particle sizes and morphology of Cu(II)-MP-bis(AMP)@boehmite were compared to the fresh catalyst. As shown, the size and morphology of recovered and reused catalyst indicated an excellent similarly to the fresh catalyst.

The heterogeneity of Cu(II)-MP-bis(AMP)@boehmite was authenticated by the hot filtration experiment. In order to this issue, the coupling reaction of Ph-O with Ph-I using Cu(II)-MP-bis(AMP)@boehmite catalyst was started and it was stopped after 30 min. In this step, 48% of biphenyl ether was formed. Then, the selected reaction was repeated and the catalyst was pick up after 30 min and the solution was permitted to proceed for 30 min again without catalyst. In this step, 51% of biphenyl ether product was obtained. it means that Cu(II)-MP-bis(AMP)@boehmite catalyst have heterogeneous nature and C–O coupling reactions are take place following heterogeneous conditions.

## Experimental

### Preparation of catalyst

Modified BNPs with (3-chloropropyl)trimethoxysilane (CPTMS@boehmite) was prepared matching to last reported method^15^. Also, MP-bis(AMP) was synthesized from condensation of 2-hydroxy benzaldehyde with 4,6-diaminopyrimidine-2-thiol^14^. Then, CPTMS@boehmite (1.0 g) was blended with MP-bis(AMP) (3 mmol) and stirred in reflux conditions of toluene for 72 h and MP-bis(AMP) ligand was attached on modified BNPs. The outcome solid (MP-bis(AMP)@boehmite) was centrifuged, washed via EtOH and dried at room temperature. Finally, for the preparation of the catalyst (Cu(II)-MP-bis(AMP)@boehmite), the MP-bis(AMP)@boehmite (0.5 g) was diffused in EtOH and blended with 1.0 mmol of Cu(NO_3_)_2_·9H_2_O. Then, the afforded mixture was stirred at 80 °C for 20 h.

### Aromatic ethers formation catalyzed by Cu(II)-MP-bis(AMP)@boehmite

Aryl halide (1 mmol), phenol (1 mmol), KOH (5 mmol), and Cu(II)-MP-bis(AMP)@boehmite (30 mg, containing 2.12 mol% of Cu) were stirred in DMSO at 130 °C and the progression of the reaction was seen by TLC. After performing of the reaction, the reaction mix was make cold to room temperature. Then, the mixture was dilute with water, the remaining catalyst was cleared by ordinary filtration and washout with ethyl acetate. The filtered solution was extracted with ethyl acetate and water. The solution was dried upon Na_2_SO_4_ (2 g). Then the solvent was vaporized and pure ether derivatives were afforded.

## Conclusion

In summary, boehmite NPs have been prepared in aqueous media and then a new Schiff base Cu-complex has been stabilized on the surface of BNPs (Cu(II)-MP-bis(AMP)@boehmite). This catalyst was evidenced using SEM imaging, WDX, EDS, AAS and TGA analysis, BET method, FT-IR spectroscopy, and XRD pattern. The yields of the obtained ethers were authenticated the good performance of Cu(II)-MP-bis(AMP)@boehmite in the C–O coupling reaction toward formation of diaryl ethers. Present method is practicable for an unlimited range of aryl halide derivatives of I, Br and Cl containing other functional groups with electron-withdrawing or electron-donating nature. Excellent stability and heterogeneous nature of Cu(II)-MP-bis(AMP)@boehmite were certified by hot filtration examination.

## Supplementary Information

Supplementary Information.

## Data Availability

Data available in article Supplementary Materiall; the data that supports the findings of this study are available in the Supplementary Material of this article.
