# Supplementary material for: Tetradentate copper complex supported on boehmite nanoparticles as an efficient and heterogeneous reusable nanocatalyst for the synthesis of diaryl ethers
Source: Sci Rep. 2022 Jul 8;12:11660. doi: 10.1038/s41598-022-15921-0 (PMC9270415; doi:10.1038/s41598-022-15921-0)
Supplement: Supplementary file 1 — Supplementary Information. [file 41598_2022_15921_MOESM1_ESM.doc]

Supplementary Data

**Tetradentate copper complex supported on boehmite nanoparticles as an efficient and heterogeneous reusable nanocatalyst for the synthesis of diaryl ethers**

**Arida Jabbaria^a^, Parisa Moradi^b^, Maryam Hajjami^c^, Bahman Tahmasbi^b^**

*^a^ Department of Chemistry, Qeshm Branch, Islamic Azad University, Qeshm, Iran. E-mail address: arida_jabbari@yahoo.com*

*^b^Department of Chemistry, Ilam University, P.O. Box 69315516, Ilam, Iran.*

*^c^ Department of Organic Chemistry, Faculty of Chemistry, Bu-Ali Sina University, 6517838683, Hamedan, Iran.*

In this work boehmite nanoparticles (BNPs) were prepared through addition of aqueous solution of NaOH to solution of Al(NO_3_)_3_.9H_2_O. Then, the surface of BNPs was modified by (3-chloropropyl)trimethoxysilane (CPTMS) and further tetradentate ligand (MP-bis(AMP)) was anchored on its surface. At final step, a tetradentate organometallic complex of copper was stabilized on the surface of modified BNPs (Cu(II)-MP-bis(AMP)@boehmite). These obtained nanoparticles were characterized using SEM imaging, WDX, EDS, AAS and TGA analysis, BET method, FT-IR spectroscopy, and XRD pattern. In continue, the catalytic activity of Cu(II)-MP-bis(AMP)@boehmite has been used as a much efficient, reusable and hybrid of organic-inorganic nanocatalyst in the synthesis of ether derivatives through C-O coupling reaction under palladium-free and phosphine-free conditions. Cu(II)-MP-bis(AMP)@boehmite catalyst has been recovered and reused again for several times in the synthesis of ether derivatives. To prove the successful synthesis of ethers, several selected products from ethers were identified by ^1^H NMR spectroscopy (Supplementary data, Figure S1 and S2).

**Key words:** Boehmite nanoparticles; coupling reaction; ether derivatives; copper complex; C-O coupling reactions.

**1-methoxy-3-phenoxybenzene:** ^1^H NMR (400 MHz, CDCl_3_): δ_H_= 7.41-7.35 (t, *J*= 12 Hz, 2H), 7.30-7.24 (t, *J*= 16 Hz, 1H), 7.17-7.05 (m, 3H), 6.71-6.59 (m, 3H), 3.82 (s, 3H) ppm (Figure S1).


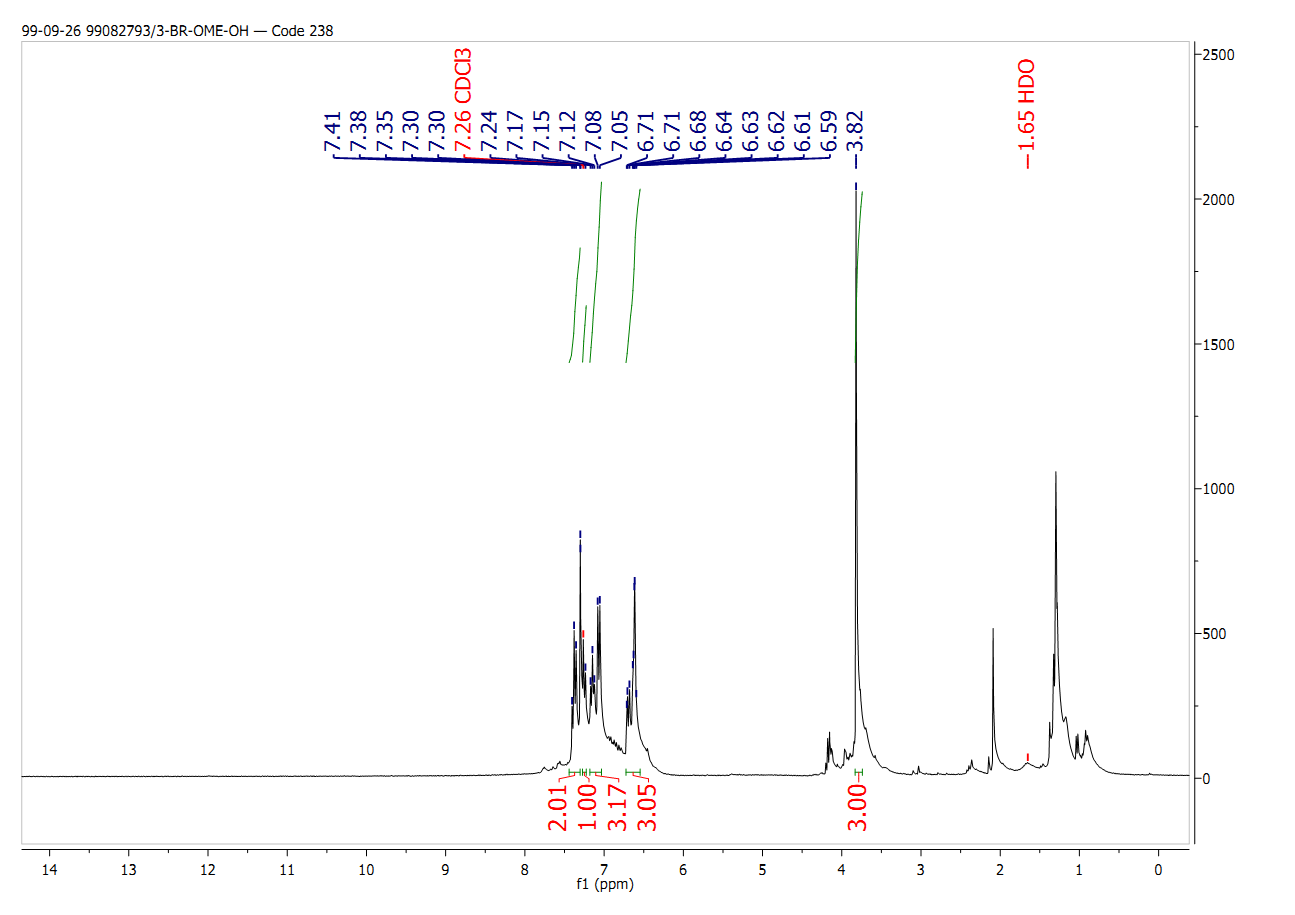


**Figure S1.** ^1^H NMR spectrum of synthesized 1-methoxy-3-phenoxybenzene

**1-nitro-4-phenoxybenzene:** ^1^H NMR (400 MHz, CDCl_3_): δ_H_= 8.19-7.16 (d, *J*= 12 Hz, 2H), 7.37-7.29 (m, 2H), 7.09-7.06 (*d, J*= 12 Hz,, 2H), 6.98-6.91 (m, 3H) ppm (Figure S2).


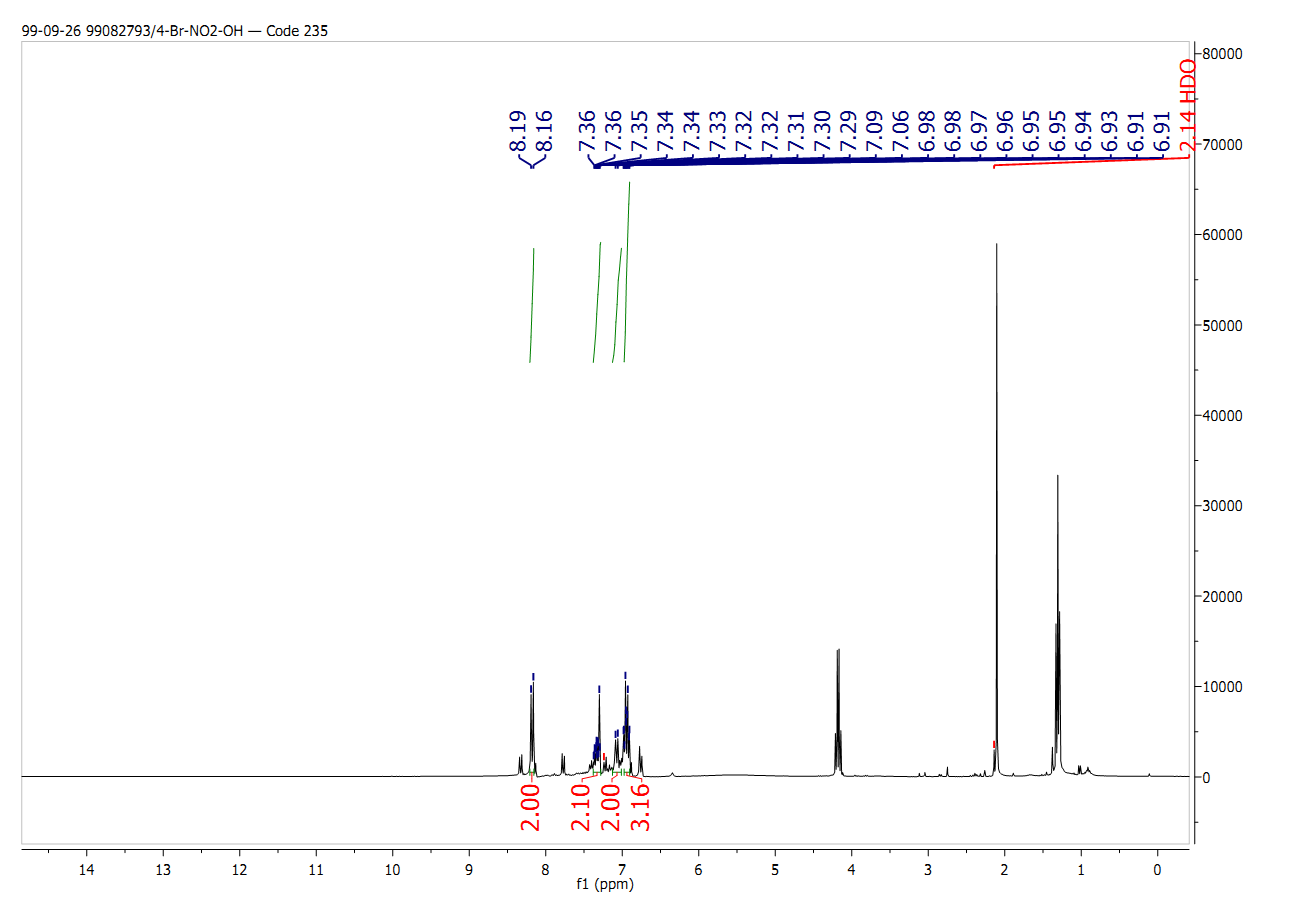


**Figure S2.** ^1^H NMR spectrum of synthesized 1-nitro-4-phenoxybenzene
